# Optimization of Aeration and Agitation Rate for Lipid and Gamma Linolenic Acid Production by *Cunninghamella bainieri* 2A1 in Submerged Fermentation Using Response Surface Methodology

**DOI:** 10.1155/2014/280146

**Published:** 2014-12-31

**Authors:** Normah Saad, Peyman Abdeshahian, Mohd Sahaid Kalil, Wan Mohtar Wan Yusoff, Aidil Abdul Hamid

**Affiliations:** ^1^School of Biosciences and Biotechnology, Faculty of Science and Technology, Universiti Kebangsaan Malaysia, 43600 Bangi, Selangor, Malaysia; ^2^Department of Chemical and Process Engineering, Faculty of Engineering and Built Environment, Universiti Kebangsaan Malaysia, 43600 Bangi, Selangor, Malaysia

## Abstract

The locally isolated filamentous fungus *Cunninghamella bainieri* 2A1 was cultivated in a 5 L bioreactor to produce lipid and gamma-linolenic acid (GLA). The optimization was carried out using response surface methodology based on a central composite design. A statistical model, second-order polynomial model, was adjusted to the experimental data to evaluate the effect of key operating variables, including aeration rate and agitation speed on lipid production. Process analysis showed that linear and quadratic effect of agitation intensity significantly influenced lipid production process (*P* < 0.01). The quadratic model also indicated that the interaction between aeration rate and agitation speed had a highly significant effect on lipid production (*P* < 0.01). Experimental results showed that a lipid content of 38.71% was produced in optimum conditions using an airflow rate and agitation speed of 0.32 vvm and 599 rpm, respectively. Similar results revealed that 0.058 (g/g) gamma-linolenic acid was produced in optimum conditions where 1.0 vvm aeration rate and 441.45 rpm agitation rate were used. The regression model confirmed that aeration and agitation were of prime importance for optimum production of lipid in the bioreactor.

## 1. Introduction

The extensive research studies have been carried out over the last decades to develop lipid production. These attempts have aimed at improving the economic production of microbial lipids rather than plant and animal derived oils. Lipids have been used as transesterified form for biofuel production. Microbial oil (single cell oil) offers favourable advantages over plant oils and animal fats such as reduced life circulation, low labour efforts, higher possibility for large scale production, and low affection by climate changes [1, 2].

Among microorganisms, Zygomycetes have shown to produce microbial oil from organic substances [3, 4]. Previous studies have revealed that a high amount of lipid could be accumulated by the fungal strains of* Cunninghamella* spp. depending on the fermentation methods and culture conditions [5, 6]. Similar studies have shown that a high lipid accumulation is attained by* Cunninghamella bainieri* 2A1 in shake flask culture [7]. It is worth noticing that microbial lipids are mostly comprised of triacylglycerols (TAGs) with the lower quantities of free fatty acids, natural lipids (monoacylglycerols, diacylglycerols, and steryl-esters), sterols, and polar fractions such as phospholipids, sphingolipids, and glycolipids. It has been found that when oleaginous microorganisms grow on the substrates with a hydrophobic characteristic the lipid accumulated contains a low quantity of TAGs. The process of lipid production from hydrophobic substrates has been known as de novo lipid accumulation. On the contrary, lipids produced from sugar-based substances show a high amount of TAGs in their compositions. Lipid synthesis from sugar-rich substrates has been called ex novo lipid accumulation [8]. A number of hydrophobic substrates have been used in the single cell oil (SCO) production by Zygomycetes including varied oils derived from vegetables (olive oils, corn oils, and sun flower oils), pure fatty-free acids, fatty esters, and fatty wastes such as crud fish oils [8, 9]. A wide range of sugar-based substances and agricultural wastes have already been utilized in de novo lipid accumulation including glycerol [10], sweet sorghum [11], rice hulls hydrolysate [12], xylose [5], orange peel [6], tomato waste hydrolysate [13], pectin [14], and corn steep [15]. Moreover, oleaginous microorganisms are capable of producing lipid from sugars-based substrate in different ways of that from fats [16].

It is well known that submerged fermentation process is affected by varied operating parameters such as incubation temperature, pH, aeration, and agitation. The scale-up of microbial product formation from a shake flask to a bioreactor involves optimization of culture conditions in fermentation processes [17]. Among operating factors, agitation and aeration are pivotal in aerobic fermentation bioreactors since they are of prime importance in industrial bioprocess and scale-up of aerobic biosynthesis systems [18]. A large number of fermentation processes have already been performed to produce microbial lipid by a wide range of fungi in a shake flask scale during the last decades [1, 3–5, 19–21]. However, much less work has been carried out to produce fungal lipid in scale-up bioreactors using* Cunninghamella* sp. The current research work aimed to study fungal lipid production by* Cunninghamella bainieri* 2A1 in an aerated submerged bioreactor. The effect of airflow rate and agitation intensity on lipid and GLA production was investigated by response surface methodology (RSM) based on a central composite design (CCD).

## 2. Materials and Methods

### 2.1. Microorganism and Inoculum Preparation

Locally isolated* Cunninghamella bainieri* 2A1 was obtained from School of Biosciences and Biotechnology, Faculty of Science and Technology, University Kebangsaan Malaysia*. Cunninghamella bainieri* 2A1 was maintained on potato dextrose agar (PDA) at 4°C. Inoculum preparation was carried out using spore suspension including 10^5^ spores/mL harvested from 7-day-old PDA plates. Seed culture was prepared by transferring 20 mL of spore suspension into a 500 mL conical flask containing 180 mL of nitrogen-limited medium (Kendrick medium). Seed culture was then incubated at 30°C with an agitation rate of 250 rpm for 48 h and kept for the inoculation of culture medium.

### 2.2. Culture Medium and Fermentation in Bioreactor

The Kendrick medium [22] was used in this study. The composition of this medium was as follows (in g/L): glucose, 30; (NH_4_)_2_C_4_H_4_O_6_, 1.0; KH_2_PO_4_, 7.0; Na_2_HPO_4_, 2.0; MgSO_4_·7H_2_O, 1.5; CaCl_2_·2H_2_O, 0.1; FeCl_3_·6H_2_O, 0.008; ZnSO_4_·7H_2_O, 0.001; CuSO_4_·5H_2_O, 0.001; Co (NO_3_)_2_·6H_2_O, 0.0001; MnSO_4_·5H_2_O, 0.0001; and yeast extract, 1.5. The Kendrick medium was then transferred into a 5 L submerged bioreactor with a working volume 4 L. The initial pH of the culture medium was adjusted to 6.0 using 1.0 M HCl or 1.0 M NaOH. The bioreactor was sterilized at 121°C for 30 min. Seed culture with an inoculums size of 10% (v/v) was aseptically added to the medium. The batch fermentation was carried out at 30°C for 120 h in different aeration and agitation rates according to the experimental design.

### 2.3. Analytical Methods

The fungal mycelium was harvested by the filtration of 100 mL of culture suspension using filter paper (Whatman No. 1). The filtered mycelium was washed with 200 mL of distilled water, stored at −20°C for 24 h, and then put under freeze-dried conditions (Shell Freeze Dry, Labconco Lyph. Lock 6) for 24 h to obtain the dry weight. The dry weight of fungal cells was determined using a balance and it was used to determine the biomass, lipid, and GLA. A mixture of chloroform and methanol with a ratio of 2 : 1 (v/v) was then added to dried mycelia, mixed, and followed by the filtration of mixture to extract lipid. Extracted lipid was transesterified with 5% sodium methoxide methanol solution [23]. The chloroform layer was obtained and evaporated using a rotary evaporator. The resultant colorless or pale-yellow transparent methyl esters were analyzed by a Shimadzu GC-14A gas chromatography equipped with a 3.1 m glass column of 3.2 mm bore packed (Shimadzu Shinchrom E71 5%/Shimalite 80–100). The instrument was fitted with a flame ionization detector. Identification of peaks was based on standards from* Orbiting Scientific Technology Sdn Bhd*. Fungal biomass was expressed as gram per liter of culture medium (g/L). Lipid content was defined as the percentage of gram of lipid produced per gram of biomass (%). GLA content was defined as the gram of GLA produced per gram of lipid-free biomass (g/g).

### 2.4. Experimental Design

A series of experiments was designed based on a central composite design (CCD) for two independent variables, and each variable varied at five levels. The experimental variables studied were agitation rate (rpm) and aeration rate (vvm). Each variable was coded at five levels of −1.41, −1, 0, +1, and +1.41. The coded values and the actual levels of the variables are given in Table 1. The design matrix of the performed experimental runs is shown in Table 2 representing thirteen treatment combinations of batch culture.

### 2.5. Statistical Model

Experimental data from the mixture design (Table 2) were used to fit a second-order polynomial regression model (1) on experimental results (Table 2) to represent product formation as a function of variables tested:

(1) $$\begin{array}{c} Y=a_{0}+\sum a_{i}X_{i}+\sum a_{ii}X_{i}^{2}+\sum a_{ij}X_{i}X_{j}, \end{array}$$

where *Y* is the measured response, *X*_*i*_ and *X*_*j*_ are the independent variables, **a**_0_ represents the intercept, and **a**_*i*_, **a**_*ii*_, and **a**_*ij*_ are the regression coefficients of the model [24]. The behavior of the generated model for two independent variables is expressed mathematically as

(2) $$\begin{array}{c} Y=a_{0}+a_{1}X_{1}+a_{2}X_{2}+a_{11}X_{1}^{2}+a_{22}X_{2}^{2}+a_{12}X_{1}X_{2}, \end{array}$$

where *Y* is the measured response, **a**_1_ and **a**_2_ are linear coefficients, **a**_11_ and **a**_22_ are squared coefficients, and **a**_12_ is an interaction coefficient. *X*_1_ and *X*_2_ represent coded values of aeration rate (vvm) and agitation speed (rpm), respectively. Three responses were studied including biomass concentration (g/L), lipid content (%), and GLA content (g/g). Statistical analysis of the data was performed using Design-Expert software (version 6.0.6 Stat-Ease, Inc.). The same software was used for optimization of the variables.

### 2.6. Verification of the Quadratic Model

In order to validate optimum conditions predicted by the empirical model, a set of batch fermentation was performed in the bioreactor under optimum conditions.

## 3. Results and Discussion

### 3.1. Lipid and GLA Production in the Bioreactor

Experimental results of lipid and GLA production by* Cunninghamella bainieri* 2A1 according to CCD are shown in Table 2. As can be observed, two independent variables (aeration rate and agitation intensity) were controlled at the levels determined by experimental design, which were represented as −1.41, −1, 0, 1, and 1.41 (Table 1). As can be seen from results in Table 2, treatments 2, 4, 6, 8, and 11 included center points of design in which same aeration rate and agitation intensity were used for the estimation of test error. As can be found, treatment 7 showed high levels of lipid and GLA concentrations with values as high as 4.74 g/L and 747.72 mg/L, respectively, where aeration and agitation rates were set at 1.14 vvm (1.41 as a coded value) and 400 rpm (0 as a coded value). Lipid accumulation by different fungi such as* Cunninghamella echinulata*,* Mortierella isabellina*, and* Mucor rouxii* have previously been studied [5, 6, 10–12]. In line with this study, Fakas et al. [13] showed that flask culture of* Cunninghamella echinulata* on a tomato waste hydrolysate medium could produce a lipid concentration of 7.8 g/L. The production of SCO by* Cunninghamella echinulata* and* Mortierella isabellina* was studied in shake-flask culture using sugar-based medium. The experimental results revealed that* C. echinulata* produced 3.9 g/L lipid and 760.5 mg/L GLA; however,* M. isabellina* triggered 9.9 g/L lipid and 346.5 mg/L GLA [25]. The same study revealed that lipid biosynthesis by* M. isabellina* from glucose in a 3-L bioreactor increased up to 12.7 g/L. In this regard, studies fulfilled by Papanikolaou et al. [26] showed that* Thamnidium elegans* CCF-1465 produced a lipid concentration of 9 g/L in shake flask using culture medium containing a mixture of glucose, fructose, and sucrose. On the other hand, the biosynthesis of lipid by* Thamnidium elegans* from glucose in a 3-L submerged bioreactor exhibited a lipid and GLA concentration of 13.9 g/L and 742 mg/L, respectively, after 200 h fermentation [27]. It was noted that* M. isabellina* could produce 301 mg/L GLA from lactose supplemented whey [28].

### 3.2. Biomass Concentration in the Bioreactor

**Figure 1:**
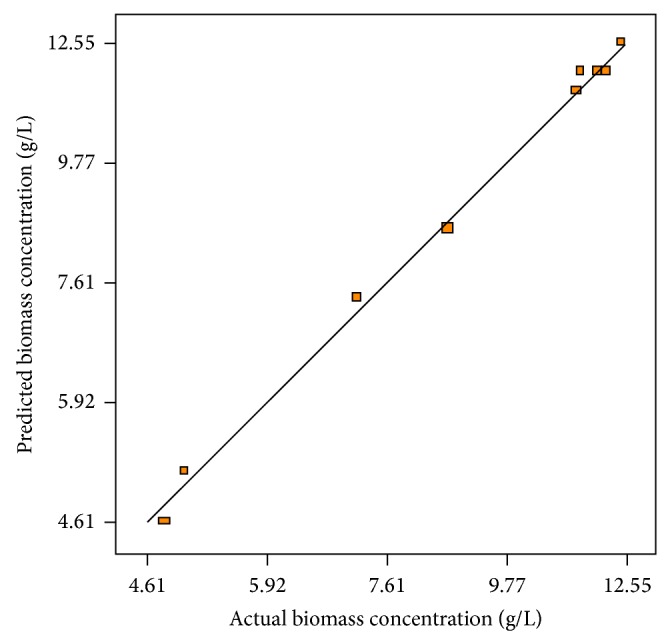
Experimental biomass concentration produced versus the predicted biomass concentration by the statistical model under operating conditions.

**Figure 2:**
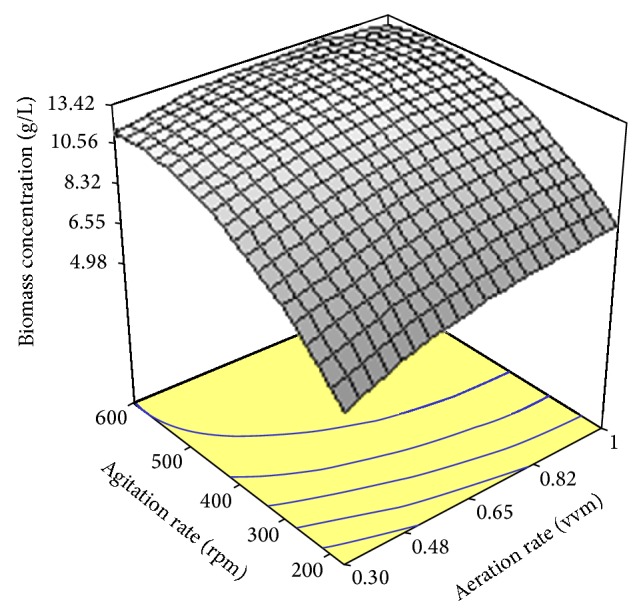
Response surface plot showing the simultaneous effects of aeration rate and agitation rate on biomass production by* Cunninghamella bainieri* 2A1.

The biomass production by* Cunninghamella bainieri* 2A1 with different combinations of aeration and agitation rate studied is shown in Table 3. As can be seen, treatments 2, 4, 6, 8, and 11 included center points in the experimental design in which the same aeration rate and agitation speed were used. As shown in Table 3, the highest value of biomass concentration was obtained in the treatment 9 with the value as high as 12.42 g/L where an aeration rate of 1.0 vvm (1 as a coded value) and an agitation rate of 600 rpm (1 as a coded value) were applied in the bioreactor. However, the lowest biomass concentration (4.80 g/L) was measured when fermentation process was carried out at an aeration rate and agitation speed of 0.65 vvm (0 as a coded value) and 117.16 rpm (−1.41 as a coded value), respectively (treatment 3). On the basis of the quadratic polynomial equation of response surface model (2), the present model and data analysis was defined for combined effects of the independent variables under study in terms of coded factors (3). The model was also transformed to fit the model on the experimental data:

(3) Y=2.47+0.11X1+0.33X2−0.081X12 −0.23X22−0.066X1X2,

where *Y* is the biomass concentration value (g/L) and *X*_1_ and *X*_2_ are the coded values of aeration rate (vvm) and agitation rate (rpm), respectively. The statistical significance of the fitted model was evaluated using the statistical test for analysis of variance (ANOVA) (Table 4). As the results shown in Table 4, calculated model's *F* value of 335.28 with a probability value (*P* > *F*) less than 0.0001 suggested that the selected quadratic model was significant and fitted well to the experimental data (*P* < 0.01). As can be observed from Table 3, the values predicted by the experimental model were also close to the actual values obtained in the experimental results. The lack of fit is a measure of the failure of a model to represent data in the experimental domain at which data points were not included in the regression model or variations in the models cannot be accounted by random error. If there is a significant lack of fit, the response is not fitted. The *F* value for lack of fit with a value of 4.16 implied that the lack of fit was insignificant and hence the model was valid for further studies. Table 4 also shows the significance of linear, interaction, and quadratic effects of the variables based on their probability values. As can be seen, linear terms (*X*_1_ and *X*_2_), interaction effect (*X*_1_ *X*_2_), and quadratic terms (*X*_1_^2^ and *X*_2_^2^) of aeration rate and agitation speed had highly significant effects on biomass concentration (*P* < 0.01). The multiple coefficient of determination (*R* ^2^) represents the measurement of the degree of the reduction in the response variability. It is postulated that *R* ^2^ value higher than 0.9 represents a stronger regression model for the prediction of the response [29]. In this case, *R* ^2^ with the acceptable value of 0.9958 implied that 99.58% of the variability in the response could be attributed to the independent parameters studied and only 0.42% of the total variation could not be explained by the quadratic model. A regression model can be used to predict observations on the response (biomass concentration) corresponding to particular values of the regressor variables.  Figure 1 shows observed biomass production versus those predicted from the model (3). This figure confirms that the predicted data of the response surface by the statistical model were consistent with the observed ones in the range of the operating variables. In order to find out the interaction effects between variables on the response, three-dimensional response surface graph was constructed by plotting the response on the *z*-axis against two independent variables (Figure 2).

Figure 2 shows the combined effect of aeration rate and agitation speed on the biomass concentration. As can be seen, an increase in biomass concentration occurred when aeration rate began to increase with the low level of agitation speed. A subsequent rise in agitation rate resulted in a significant increase in biomass concentration which indicated an interaction between aeration and agitation affecting biomass production.

**Figure 3:**
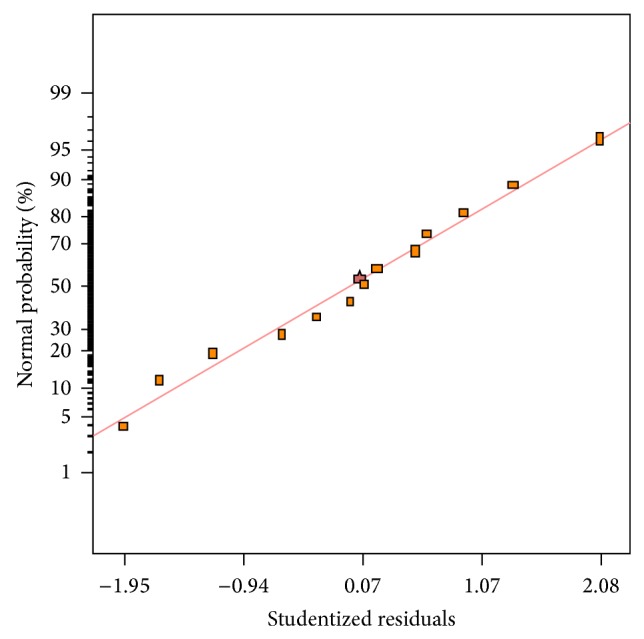
Normal probability of studentized residuals for biomass concentration

**Figure 4:**
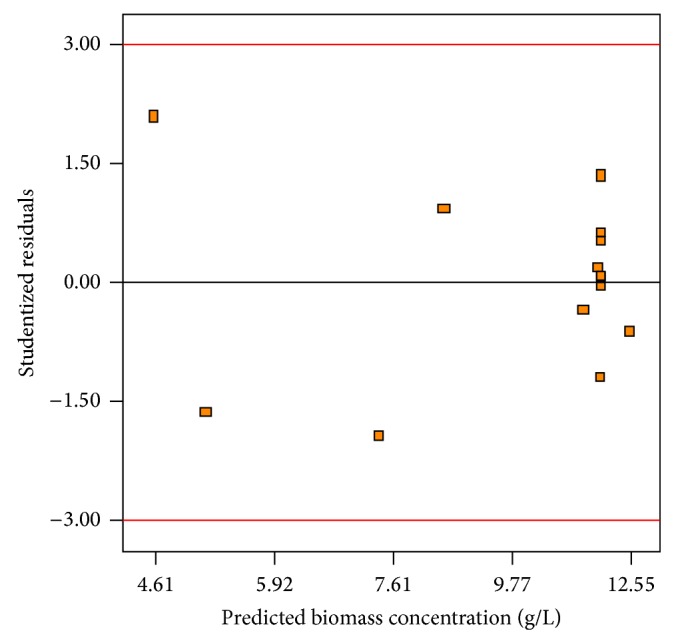
Plot of studentized residuals versus the predicted biomass concentration.

Generally, it is important to find out the adequacy of the fitted model to make sure that it provides an adequate approximation to the real conditions. By plotting a normal probability of residuals, a check was made for the normality assumption (Figure 3). As can be seen from Figure 3, the normal assumption was acceptable as a normal distribution of residuals was generated. A plot of residuals versus the predicted response values is given in Figure 4. As shown, there was a random scatter of residuals across the graph which suggested that the variance of original observations was constant for all values of *y*-axis. Both of the plots (Figures 3 and 4) confirmed that the empirical model was adequate to describe biomass production by the response surface. The empirical model was also used to determine optimum conditions for the highest production of biomass. The optimum conditions suggested by the quadratic model were an aeration rate of 0.63 vvm and an agitation rate of 506 rpm. The biomass production of 13.07 g/L was predicted by the regression model in optimum conditions suggested. In order to verify optimum conditions, a fermentation experiment was run by the cultivation of* Cunninghamella bainieri* 2A1 in the bioreactor under optimum conditions determined. The experimental result showed that a biomass concentration of 13.0 g/L was obtained confirming a high fit between the statistical model and experimental data. It was noted that the cultivation of* Thamnidium elegans* in the bioreactor using glucose-rich medium brought about 30.1 g/L biomass [27]. Fakas et al. [5] found that* Cunninghamella echinulata* and* Mortierella isabellina* produced a biomass concentration of 15 g/L and 27 g/L, respectively, in shake flask containing glucose-based medium. In an attempt for the production of lipid in a laboratory-scale bioreactor with a working volume of 2.5 L, the highest biomass produced by* Mortierella ramanniana*,* Mucor* sp., and* Zygorhynchus moelleri* on glycerol were 7.2 g/L, 1.6 g/L, and 0.7 g/L, respectively [10]. The cultivation of oleaginous strain* Thamnidium elegans* in the bioreactor containing glucose and olive mill wastewater resulted in the production of 2.5 g/L of fat-free biomass [30].

### 3.3. Lipid Content in the Bioreactor

Lipid content (lipid percentage) produced by* Cunninghamella bainieri* 2A1 at varied combinations of aeration rate and agitation speed is shown in Table 3. As can be seen, the lipid content obtained in the center points of experimental design is presented in treatments 2, 4, 6, 8, and 11 using an aeration rate and agitation speed of 0.65 vvm (0 as a coded value) and 400 rpm (0 as a coded value), respectively. As can be found, the highest lipid content was obtained in the treatment 7 with the value as high as 39.33% when fermentation process was carried out at an aeration rate of 1.14 vvm (1.41 as a coded value) and an agitation rate of 400 rpm (0 as a coded value). However, minimum lipid content of 22.5% was measured when fermentation process was carried out at an aeration rate and agitation speed of 0.30 vvm (−1 as a coded value) and 200 rpm (−1 as a coded value), respectively (treatment 13). By applying multiple regression analysis to the test results, the following second order polynomial equation (4) was obtained in order to represent lipid content as a function of airflow rate and agitation speed:

(4) Y=37.75+1.41X1+3.80X2−1.161X12 −3.86X22−3.93X1X2,

where *Y* is the lipid content (%) and *X*_1_ and *X*_2_ are the coded values of aeration rate (vvm) and agitation rate (rpm), respectively. The statistical test for analysis of variance (ANOVA) was generated to evaluate statistical significance of the empirical model selected (Table 5).

It is noteworthy that the actual values of lipid content obtained in the experimental results well matched the values predicted by the experimental model (Table 3). As can be observed from Table 5, computed model's *F* value of 13.84 with a probability value (Prob > *F*) of 0.0016 indicated that the quadratic regression model was significantly fitted for experimental data (*P* < 0.01). The lack of fit related to *F* value (3.79) implied that the lack of fit was not significant relative to the pure error. Hence, the regression model was acceptable for further evaluation. As can be seen from Table 5, the linear and quadratic term of agitation rate (*X*_2_ and *X*_2_^2^) had highly significant effect on lipid yield (*P* < 0.01). The regression model also showed that the interaction effect between aeration rate and agitation rate (*X*_1_ *X*_2_) was highly significant at 99% probability level (*P* < 0.01). The multiple coefficient of determination (*R* ^2^) with the satisfactory value of 0.9081 implied that 90.81% of the variability in response could be explained by the empirical model and only 9.19% of the total variation could not be explained by the model.

**Figure 5:**
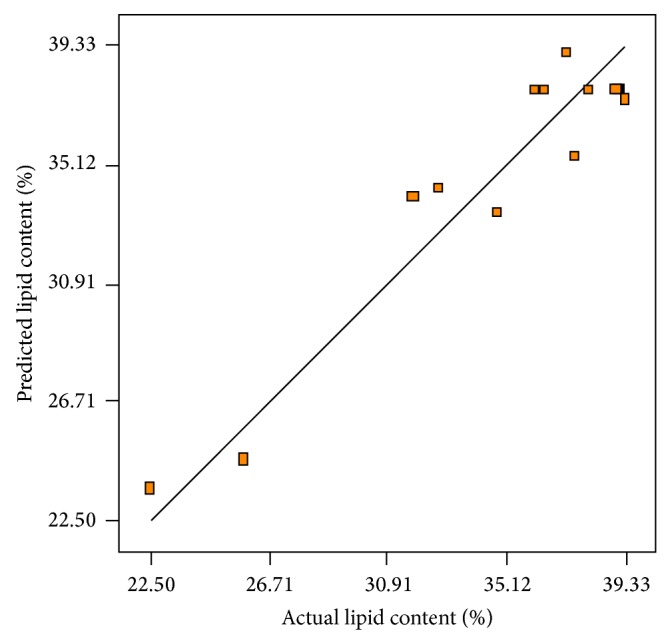
Experimental lipid content produced versus the predicted lipid content by the statistical model under operating conditions.

**Figure 6:**
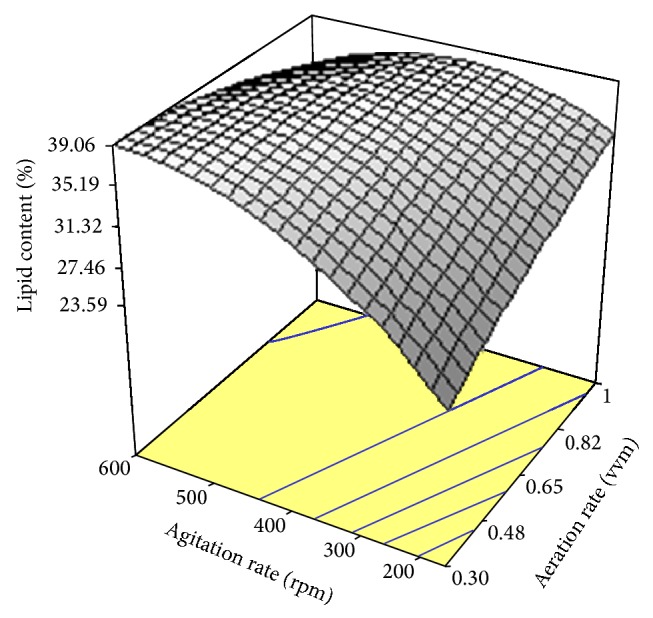
Response surface plot showing the simultaneous effects of aeration rate and agitation rate on lipid content produced by* Cunninghamella bainieri* 2A1.

Figure 5 plots experimental results of lipid content versus the values of lipid content predicted by the quadratic model (4). This figure confirms that the predicted lipid content by the regression model was in line with the obtained results in the range of the variables studied. In order to describe the effects of aeration and agitation on lipid content, three-dimensional response surface graph of simultaneous effect of the variables on lipid percentage was constructed on the basis of the model (Figure 6). As shown, the variations in aeration rate interacted with agitation rates used so that an increment in aeration rate and agitation speed concurrently increased lipid content up to a maximum level.

**Figure 7:**
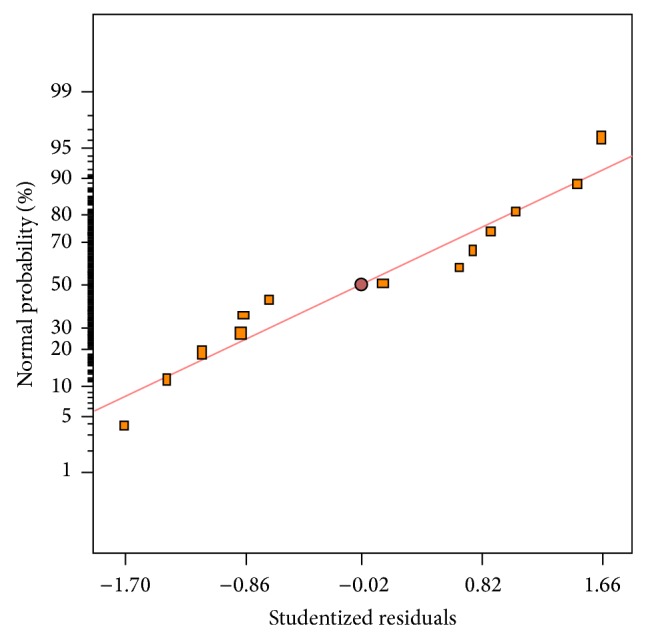
Normal probability of studentized residuals for lipid content.

**Figure 8:**
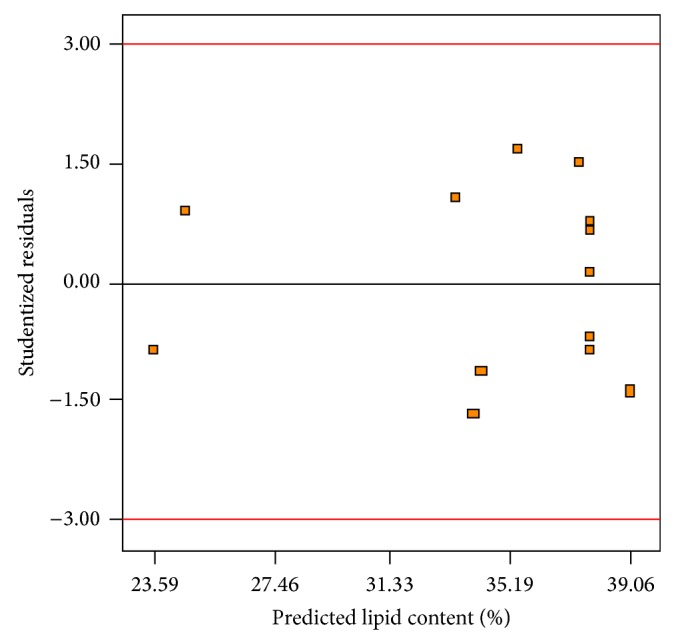
Plot of studentized residuals versus the predicted lipid content.

The normal plot of residuals for lipid content is depicted in Figure 7. As can be seen, a satisfactory distribution of normal probability in relation to residuals was generated. Moreover, the plot of residuals versus the predicted lipid yield (Figure 8) showed an acceptable scatter of residuals across the graph. The plots illustrated in Figures 7 and 8 indicated that the polynomial regression model had satisfactory adequacy for representing lipid content obtained by the response surface. The analysis of the statistical model showed that the optimum conditions for the highest achievement of lipid content was obtained when airflow and agitation speed were used at the rates of 0.32 vvm and 599 rpm, respectively. The statistical model predicted that a lipid content of 39.04% could be produced under optimum conditions suggested. A verification experiment was performed in optimum conditions to measure lipid percentage in the bioreactor. Experimental result showed that a lipid content of 38.71% was produced which confirmed that the model used was valid and the results could be reproducible. Scaling-up of lipid production by* M. isabellina* on glucose in a submerged-bioreactor showed that a lipid content of 72.5% was produced [25]. Submerged fermentation of* Thamnidium elegans* CCF-1465 in shake-flask culture revealed that this strain assimilated sugars in the medium and produced 70% lipid in dry biomass [26]. The production of lipid by* Mucor circinelloides* from citric acid as sole carbon source was studied by Aggelis [31]. This study revealed that maximum 15.9% lipid content was obtained where 10 g/L citric acid was supplemented to the culture medium.

### 3.4. GLA Content in the Bioreactor

Table 3 shows the experimental results of GLA content obtained from the cultivation of* C. bainieri* 2A1 in different levels of aeration and agitation rates which were determined by the experimental design. It is obvious that the center points of the experimental design were in treatments 2, 4, 6, 8, and 11. As is evident, the highest value of GLA content was obtained in the treatment 7 with the value as high as 0.062 (g/g) when fermentation process was carried out at an aeration rate of 1.14 vvm (1.41 as a coded value) and an agitation rate of 400 rpm (0 as a coded value). However, minimum GLA content of 0.029 (g/g) was measured when fermentation process was carried out at an aeration rate and agitation speed of 0.30 vvm (−1 as a coded value) and 200 rpm (−1 as a coded value), respectively (treatment 13). A second order model equation (5) was fitted on the experimental data obtained according to the experimental design:

(5) Y=0.049+4.121E−003X1+6.432E−003X2+2.600E −003X12−5.900E−003X22−4.000E−003X1X2,

where *Y* is the GLA content (g/g) and *X*_1_ and *X*_2_ are the coded values of aeration rate (vvm) and agitation rate (rpm), respectively. The significance of the response surface quadratic model was evaluated using statistical analysis of variance (ANOVA), which is essential for determining patterns of interaction between experimental variables (Table 6). On the other hand, GLA content obtained from the experimental results was appropriately consistent with the values predicted by the experimental model (Table 3). Obviously, the model *F* value of 27.81 with a probability value (Prob > *F*) of 0.0002 implied that the regression model was significant (*P* < 0.01). Furthermore, the lack of fit of the model with the value of 3.97 indicated that the lack of fit was insignificant. Therefore, the empirical model adequately fitted on the experimental results. As shown in Table 6, the linear effect of aeration rate and agitation rate (*X*_1_ and *X*_2_) had a highly significant effect on GLA content (*P* < 0.01). Moreover, the quadratic effects of aeration rate and agitation rate (*X*_1_^2^ and *X*_2_^2^) on GLA content were significant at 95% probability level (*P* < 0.05) and 99% probability level (*P* < 0.01), respectively. The significance study of the model terms also revealed that the aeration rate and agitation rate had a significant interaction effect (*X*_1_ *X*_2_) on GLA content (*P* < 0.05). The *R*-squared of the model (*R* ^2^) with the value of 0.9521 suggested that 95.21% of the variability in the response (GLA content) could be explained by the regression model and only 4.79% of the total variation could not be explained by the quadratic model.

**Figure 9:**
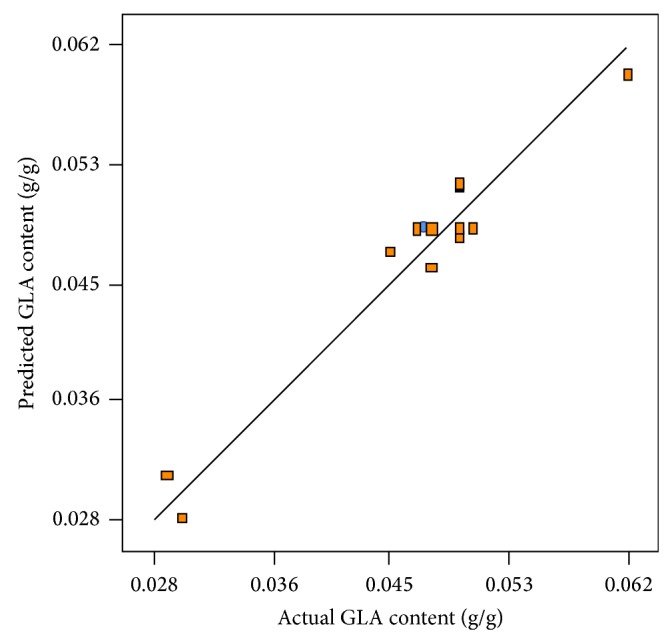
Experimental GLA content produced versus the predicted GLA content by the statistical model under operating conditions.

**Figure 10:**
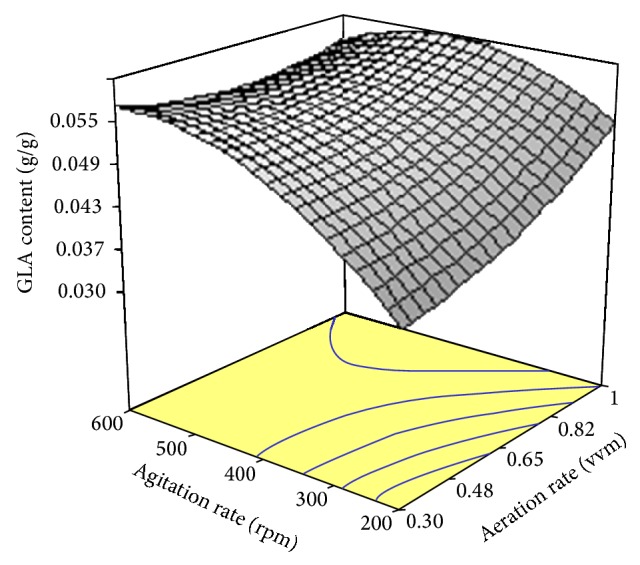
Response surface plot showing the simultaneous effects of aeration rate and agitation rate on GLA content produced by* Cunninghamella bainieri* 2A1.

**Figure 11:**
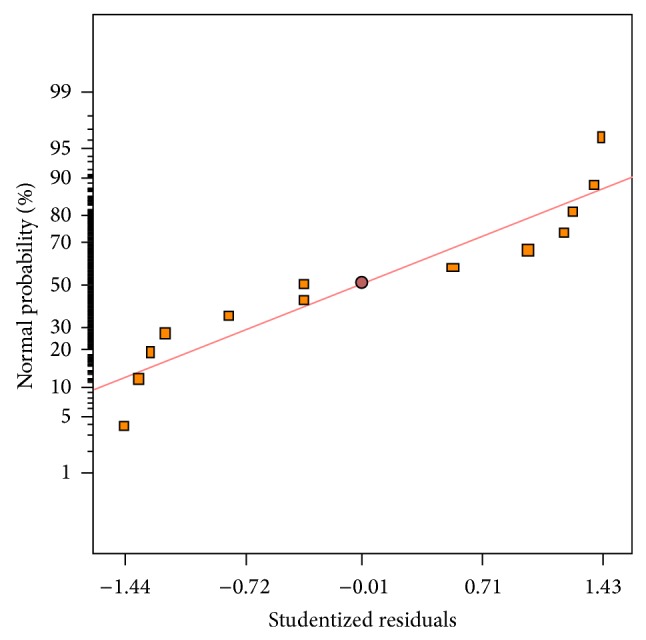
Normal probability of studentized residuals for GLA content.

**Figure 12:**
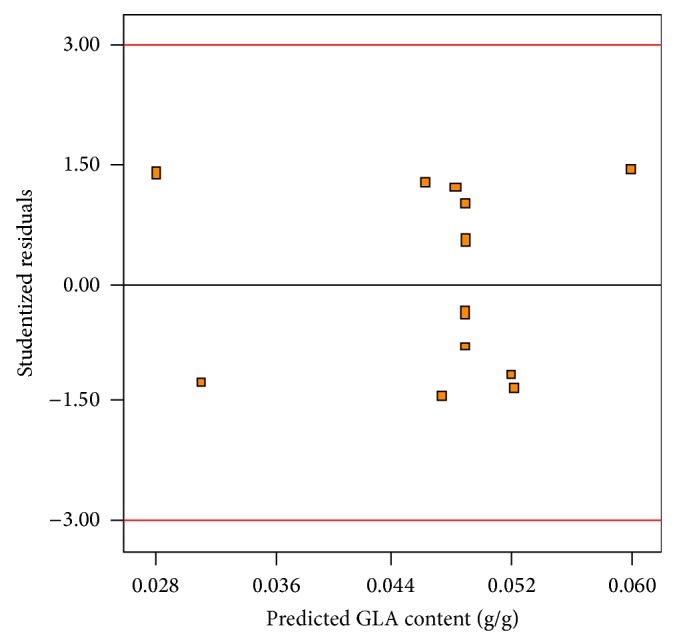
Plot of studentized residuals versus the predicted GLA content.

The values of GLA content measured in the experimental results versus predicted values by the statistical model are depicted in Figure 9. As is evident, observed GLA content values were in accordance with those predicted by the model (Table 3). The three-dimensional response surface graph of simultaneous effect of aeration and agitation was constructed to illustrate the interaction effect of these variables on GLA content (Figure 10). As can be observed, a quadratic rise in the response was formed with increased aeration and agitation rates in the range of levels tested. Hence, variations in response were affected by different rates of aeration and agitation indicating the interaction between these parameters on the GLA content produced. The normal plot of residuals for GLA content is illustrated in Figure 11. As shown, an appropriate distribution of normal probability in relation to residuals was formed. The plot of residuals versus the predicted GLA content is shown in Figure 12. Obviously, an acceptable scatter of residuals across the graph was generated. The plots shown in Figures 11 and 12 revealed that the quadratic regression model had a suitable adequacy to explain GLA content obtained by the response surface. Further statistical analysis suggested that optimum conditions for the highest production of GLA content was a flow rate of 1.0 vvm and an agitation speed of 441.45 rpm. The quadratic model also predicted that a GLA content of 0.055 (g/g) could be attained in the optimum conditions.

In order to verify optimum conditions suggested by the model, a set of experiment was performed in the optimum conditions suggested by the cultivation of* C. bainieri* 2A1 in the bioreactor under optimum conditions. The result obtained from verification experiment showed that a GLA content of 0.058 (g/g) was produced. This finding validated the accuracy of the empirical model used for attaining sufficient homogeneity and adequate aeration in the culture. It has been found that GLA synthesis is varied in relation to lipid accumulation by Zygomycetes. In this regard, the production of lipid is low at early lipid accumulation, while GLA content is high at this time. As lipid synthesis proceeds, with the increasing in the concentration of oil content the quantity of GLA starts dwindling. The process of SCO synthesis is followed by an increment in GLA content at the late process where a decrease in lipid production occurs [32].

The production of GLA by* Thamnidium elegans* on glucose in a laboratory bioreactor was investigated. It was noted that fungal strain used could trigger GLA content of 0.031 g/g [27]. The production of GLA by* Cunninghamella echinulata* and* Mortierella isabellina* in shake flask revealed that 19.5% GLA was produced by* C. echinulata* and 3.5% GLA was produced by* M. isabellina* [25]. In an attempt for GLA synthesis by different strain of Zygomycetes, it was found that GLA content produced by* Z. moelleri*,* Cunninghamella echinulata,* and* Mucor* sp., from glycerol were 21.4%, 19.9%, and 19.8%, respectively [10]. The production of lipid by* Cunninghamella echinulata* was studied by Gema et al. [6] who used orange peel as substrate for lipid synthesis. The authors found that the maximum GLA content of 1.2–15 mg/g fermented orange peel was obtained where solid-state fermentation was applied.

### 3.5. The Effect of Aeration and Agitation

Aeration has a crucial effect on SCO synthesis by oleaginous microorganisms using sugar-based medium since aeration positively increase consumption of fermentable sugars by lipid-producing microorganisms resulting in increased yield of SCO produced per unit of sugar consumed [33]. As shown in Figure 2 an increase in aeration rate from 200 vvm to 600 rpm caused a quadratic increase in biomass concentration. Similar trend was found for variations in agitation rates so that increased agitation intensities resulted in an increment in biomass production. It has been known that aeration and agitation are of great importance in aerobic culture. A rise in aeration and agitation rates leads to an increased oxygen supply, improved mixing and enhancement of mass transfer phenomenon in a bioreactor of submerged fermentation, which in turn increases microbial cell growth and biomass production [34]. As reported elsewhere, aeration rate had favorable effects on the production of GLA by* Mortierella isabellina* using olive mill wastewater [30]. Studies fulfilled by Bajaj and Singhal [35] also revealed that airflow rate and agitation speed were critical factors for biomass production by microbial cells.

Similar to biomass production, a reciprocal relation between agitation intensities and lipid yield was found (Figure 6). As can be seen, an increase in agitation rate resulted in increased lipid yield. This fact can be attributed to improved homogeneity of the culture and the enhancement of heat and nutrient transfer in the culture which increased microbial growth and metabolism-mediated product formation. In an attempt for the production of lipid by* Yarrowia lipolytica* it has been found that increased agitation intensity and aeration rate caused a rise in biomass production compared to shake flask, whereas a low lipid was accumulated compared to that in flask scale [36]. On the other hand, an increment in aeration caused a better mixing intensity with higher dissolved oxygen maintenance in the culture medium [37]. Consequently, airflow and agitation caused an interaction effect on lipid production by* C. bainieri* 2A1 (Figure 6). The model trend in the response formed for GLA yield revealed that aeration and agitation intensity had positive effects on GLA production (Figure 10) because increased aeration and agitation rates made better mechanical mixing and enhanced oxygen and nutrient transfer in the culture, which in turn a rise in mycelia growth and GLA production occurred [38].

## 4. Conclusions

This study showed a successful process modeling in the production of biomass, lipid, and GLA by* C. bainieri* 2A1 in a submerged bioreactor using RSM. The regression models fitted well with experimental results obtained. The analysis of the models revealed that airflow rates and agitation intensities had significant effects on biomass, lipid, and GLA synthesis by* C. bainieri* 2A1 in the range of operating levels determined. The product values obtained in optimum conditions were close to the values predicted by the models confirming the accuracy of the models used.

## Figures and Tables

**Table 1:** Process variables and determined levels for central composite design.

Variable	Symbol	Actual level					Coded value				
Aeration rate (vvm)	*X*_1_	0.16	0.30	0.65	1	1.14	−1.41	−1	0	1	1.41
Agitation rate (rpm)	*X*_2_	117.16	200	400	600	682.84	−1.41	−1	0	1	1.41

**Table 2:** Experimental design and test results of lipid and GLA produced by *Cunninghamella bainieri* 2A1 in the submerged bioreactor.

Run	*X*_1_	*X*_2_	Lipid concentration (g/L)	GLA concentration (mg/L)
1	−1	1	4.23	567.5
2	0	0	4.53	573.12
3	0	−1.41	1.23	144.0
4	0	0	4.60	602.82
5	1	−1	2.35	322.65
6	0	0	4.18	539.09
7	1.41	0	4.74	747.72
8	0	0	4.62	591.0
9	1	1	3.95	621.0
10	−1.41	0	3.01	433.5
11	0	0	4.31	573.12
12	0	1.41	4.34	567.36
13	−1	−1	1.125	145.0

*X*_1_: aeration rate (vvm); *X*_2_: agitation rate (rpm).

**Table 3:** Experimental design and test results of biomass concentration, lipid yield, and GLA yield produced by *Cunninghamella bainieri* 2A1 in the submerged bioreactor.

Run	*X*_1_	*X*_2_	Biomass concentration (g/L)		Lipid yield (%)		GLA yield (g/g)	
			Actual value	Predicted value	Actual value	Predicted value	Actual value	Predicted value
1	−1	1	11.35	11.35	37.28	39.06	0.05	0.052
2	0	0	11.94	11.82	38	37.75	0.048	0.049
3	0	−1.41	4.80	4.61	25.79	24.65	0.03	0.028
4	0	0	11.82	11.82	39	37.75	0.051	0.049
5	1	−1	7.17	7.38	32.79	34.27	0.045	0.047
6	0	0	11.47	11.82	36.48	37.75	0.047	0.049
7	1.41	0	12.06	11.82	39.33	37.43	0.062	0.060
8	0	0	11.82	11.82	39.17	37.75	0.05	0.049
9	1	1	12.42	12.55	31.85	34.01	0.05	0.052
10	−1.41	0	8.67	8.49	34.81	33.45	0.05	0.048
11	0	0	11.94	11.82	36.12	37.75	0.048	0.049
12	0	1.41	11.82	11.70	37.52	35.41	0.048	0.046
13	−1	−1	5.0	5.15	22.5	23.59	0.029	0.031

*X*_1_: aeration rate (vvm); *X*_2_: agitation rate (rpm).

**Table 4:** Analysis of variance for the quadratic polynomial model of biomass production by *Cunninghamella bainieri* 2A1.

Source	Polynomial coefficients	Sum of squares	DF	Mean square	*F* value	Prob > *F*
Model		1.39	5	0.28	335.28	<0.0001^*^
Intercept	2.47		1			
*X*_1_	0.11	0.11	1	0.11	127.19	<0.0001^*^
*X*_2_	0.33	0.87	1	0.87	1045.02	<0.0001^*^
*X*_1_^2^	−0.081	0.046	1	0.046	54.94	0.0001^*^
*X*_2_^2^	−0.23	0.38	1	0.38	461.40	<0.0001^*^
*X*_1_ *X*_2_	−0.066	0.018	1	0.018	21.18	0.0025^*^
Residual		5.805*E* − 003	7	8.293*E* − 004		
Lack of Fit		4.397*E* − 003	3	1.466*E* − 003	4.16	0.1010
Pure Error		1.408*E* − 003	4	3.521*E* − 004		

^*^Statistically significant at 99% probability level.

*X*_1_: aeration rate (vvm); *X*_2_: agitation rate (rpm); *X*_1_^2^ and *X*_2_^2^: the quadratic terms; *X*_1_ *X*_2_: the interaction term.

*R* ^2^ = 0.9958.

**Table 5:** Analysis of variance for the quadratic polynomial model of lipid content produced by *Cunninghamella bainieri* 2A1.

Source	Polynomial coefficients	Sum of squares	DF	Mean square	*F* value	Prob > *F*
Model		300.21	5	60.4	13.84	0.0016^*^
Intercept	37.75		1			
*X*_1_	1.41	15.83	1	15.83	3.65	0.0978
*X*_2_	3.80	115.74	1	115.74	26.68	0.0013^*^
*X*_1_^2^	−1.161	9.30	1	9.30	2.14	0.1865
*X*_2_^2^	−3.86	103.86	1	103.86	23.94	0.0018^*^
*X*_1_ *X*_2_	−3.93	61.78	1	61.78	14.24	0.0070^*^
Residual		30.37	7	4.34		
Lack of fit		22.46	3	7.49	3.79	0.1156
Pure error		7.91	4	1.98		

^*^Statistically significant at 99% probability level.

*X*_1_: aeration rate (vvm); *X*_2_: agitation rate (rpm); *X*_1_^2^ and *X*_2_^2^: the quadratic terms; *X*_1_ *X*_2_: the interaction term.

*R* ^2^ = 0.9081.

**Table 6:** Analysis of variance for the quadratic polynomial model of GLA content produced by *Cunninghamella bainieri* 2A1.

Source	Polynomial coefficients	Sum of squares	DF	Mean square	*F* value	Prob > *F*
Model		8.534*E* − 004	5	1.707*E* − 004	27.81	0.0002^**^
Intercept	0.49					
*X*_1_	4.121*E* − 003	1.359*E* − 004	1	1.359*E* − 004	22.14	0.0022^**^
*X*_2_	6.432*E* − 003	3.310*E* − 004	1	3.310*E* − 004	53.93	0.0002^**^
*X*_1_^2^	2.600*E* − 003	4.703*E* − 005	1	4.703*E* − 005	7.66	0.0278^*^
*X*_2_^2^	−5.900*E* − 003	2.422*E* − 004	1	2.422*E* − 004	39.46	0.0004^**^
*X*_1_ *X*_2_	−4.000*E* − 003	6.400*E* − 005	1	6.400*E* − 005	10.43	0.0145^*^
Residual		4.295*E* − 005	7	6.136*E* − 006		
Lack of fit		3.215*E* − 005	3	1.072*E* − 005	3.97	0.1081
Pure error		1.080*E* − 005	4	2.700*E* − 006		

^*^Statistically significant at 95% probability level.

^**^Statistically significant at 99% probability level.

*X*_1_: aeration rate (vvm); *X*_2_: agitation rate (rpm); *X*_1_^2^ and *X*_2_^2^: the quadratic terms; *X*_1_ *X*_2_: the interaction term.

*R* ^2^ = 0.9521.

## References

[1] Abdeshahian P., Dashti M. G., Kalil M. S., Yusoff W. M. W. (2010). Production of biofuel using biomass as a sustainable biological resource. *Biotechnology*.

[2] Li Q., Du W., Liu D. (2008). Perspectives of microbial oils for biodiesel production. *Applied Microbiology and Biotechnology*.

[3] Venkata Subhash G., Venkata Mohan S. (2014). Lipid accumulation for biodiesel production by oleaginous fungus *Aspergillus awamori*: influence of critical factors. *Fuel*.

[4] Dey P., Banerjee J., Maiti M. K. (2011). Comparative lipid profiling of two endophytic fungal isolates—*Colletotrichum* sp. and *Alternaria* sp. having potential utilities as biodiesel feedstock. *Bioresource Technology*.

[5] Fakas S., Papanikolaou S., Batsos A., Galiotou-Panayotou M., Mallouchos A., Aggelis G. (2009). Evaluating renewable carbon sources as substrates for single cell oil production by *Cunninghamella echinulata* and *Mortierella isabellina*. *Biomass and Bioenergy*.

[6] Gema H., Kavadia A., Dimou D., Tsagou V., Komaitis M., Aggelis G. (2002). Production of *γ*-linolenic acid by *Cunninghamella echinulata* cultivated on glucose and orange peel. *Applied Microbiology and Biotechnology*.

[7] Taha E. M., Omar O., Yusoff W. M. W., Hamid A. A. (2010). Lipid biosynthesis in *Cunninghamella bainieri* 2A1 in N-limited and N-excess media. *Annals of Microbiology*.

[8] Papanikolaou S., Aggelis G. (2011). Lipids of oleaginous yeasts. Part I: biochemistry of single cell oil production. *European Journal of Lipid Science and Technology*.

[9] Bati N., Hammond E. G., Glatz B. A. (1984). Biomodification of fats and oils: trials with Candida lipolytica. *Journal of the American Oil Chemists' Society*.

[10] Bellou S., Moustogianni A., Makri A., Aggelis G. (2012). Lipids containing polyunsaturated fatty acids synthesized by zygomycetes grown on glycerol. *Applied Biochemistry and Biotechnology*.

[11] Economou C. N., Aggelis G., Pavlou S., Vayenas D. V. (2011). Modeling of single-cell oil production under nitrogen-limited and substrate inhibition conditions. *Biotechnology and Bioengineering*.

[12] Economou C. N., Aggelis G., Pavlou S., Vayenas D. V. (2011). Single cell oil production from rice hulls hydrolysate. *Bioresource Technology*.

[13] Fakas S., Galiotou-Panayotou M., Papanikolaou S., Komaitis M., Aggelis G. (2007). Compositional shifts in lipid fractions during lipid turnover in *Cunninghamella echinulata*. *Enzyme and Microbial Technology*.

[14] Papanikolaou S., Galiotou-Panayotou M., Fakas S., Komaitis M., Aggelis G. (2007). Lipid production by oleaginous *Mucorales* cultivated on renewable carbon sources. *European Journal of Lipid Science and Technology*.

[15] Fakas S., Čertik M., Papanikolaou S., Aggelis G., Komaitis M., Galiotou-Panayotou M. (2008). *γ*-Linolenic acid production by *Cunninghamella echinulata* growing on complex organic nitrogen sources. *Bioresource Technology*.

[16] Aggelis G., Sourdis J. (1997). Prediction of lipid accumulation-degradation in oleaginous micro-organisms growing on vegetable oils. *Antonie van Leeuwenhoek*.

[17] Thiry M., Cingolani D. (2002). Optimizing scale-up fermentation processes. *Trends in Biotechnology*.

[18] Bandaiphet C., Prasertsan P. (2006). Effect of aeration and agitation rates and scale-up on oxygen transfer coefficient, *k*La in exopolysaccharide production from *Enterobacter cloacae* WD7. *Carbohydrate Polymers*.

[19] Gao D., Zeng J., Zheng Y., Yu X., Chen S. (2013). Microbial lipid production from xylose by *Mortierella isabellina*. *Bioresource Technology*.

[20] Somashekar D., Venkateshwaran G., Sambaiah K., Lokesh B. R. (2003). Effect of culture conditions on lipid and gamma-linolenic acid production by mucoraceous fungi. *Process Biochemistry*.

[21] Ruan Z., Zanotti M., Wang X., Ducey C., Liu Y. (2012). Evaluation of lipid accumulation from lignocellulosic sugars by *Mortierella isabellina* for biodiesel production. *Bioresource Technology*.

[22] Kendrick A., Ratledge C. (1992). Desaturation of polyunsaturated fatty acids in *Mucor circinelloides* and the involvement of a novel membrane-bound malic enzyme. *European Journal of Biochemistry*.

[23] Folch J., Lees M., Sloane-Stanley G. H. (1957). A simple method for the isolation and purification of total lipides from animal tissues. *The Journal of Biological Chemistry*.

[24] Dashti M. G., Abdeshahian P., Yusoff W. M. W., Kalil M. S., Hamid A. A. (2014). Repeated batch fermentation biotechnology for the biosynthesis of lipid and gamma-linolenic acid by *Cunninghamella bainieri* 2A1. *BioMed Research International*.

[25] Chatzifragkou A., Fakas S., Galiotou-Panayotou M., Komaitis M., Aggelis G., Papanikolaou S. (2010). Commercial sugars as substrates for lipid accumulation in *Cunninghamella echinulata* and *Mortierella isabellina* fungi. *European Journal of Lipid Science and Technology*.

[26] Papanikolaou S., Diamantopoulou P., Chatzifragkou A., Philippoussis A., Aggelis G. (2010). Suitability of low-cost sugars as substrates for lipid production by the fungus thamnidium elegans. *Energy and Fuels*.

[27] Zikou E., Chatzifragkou A., Koutinas A. A., Papanikolaou S. (2013). Evaluating glucose and xylose as cosubstrates for lipid accumulation and *γ*-linolenic acid biosynthesis of *Thamnidium elegans*. *Journal of Applied Microbiology*.

[28] Vamvakaki A.-N., Kandarakis I., Kaminarides S., Komaitis M., Papanikolaou S. (2010). Cheese whey as a renewable substrate for microbial lipid and biomass production by Zygomycetes. *Engineering in Life Sciences*.

[29] Vijayaraghavan P., Vincent S. G. P. (2014). Medium optimization for the production of fibrinolytic enzyme by *Paenibacillus* sp. IND8 using response surface methodology. *The Scientific World Journal*.

[30] Bellou S., Makri A., Sarris D. (2014). The olive mill wastewater as substrate for single cell oil production by Zygomycetes. *Journal of Biotechnology*.

[31] Aggelis G. (1996). Two alternative pathways for substrate assimilation by Mucor circinelloides. *Folia Microbiologica*.

[32] Fakas S., Makri A., Mavromati M., Tselepi M., Aggelis G. (2009). Fatty acid composition in lipid fractions lengthwise the mycelium of *Mortierella isabellina* and lipid production by solid state fermentation. *Bioresource Technology*.

[33] Ratledge C., Wynn J. P. (2002). The biochemistry and molecular biology of lipid accumulation in oleaginous microorganisms. *Advances in Applied Microbiology*.

[34] Sitanggang A. B., Wu H.-S., Wang S. S., Ho Y.-C. (2010). Effect of pellet size and stimulating factor on the glucosamine production using *Aspergillus* sp. BCRC 31742. *Bioresource Technology*.

[35] Bajaj I. B., Singhal R. S. (2010). Effect of aeration and agitation on synthesis of poly(*γ*-glutamic acid) in batch cultures of *Bacillus licheniformis* NCIM 2324. *Biotechnology and Bioprocess Engineering*.

[36] Papanikolaou S., Chevalot I., Galiotou-Panayotou M., Komaitis M., Marc I., Aggelis G. (2007). Industrial derivative of tallow: a promising renewable substrate for microbial lipid, single-cell protein and lipase production by *Yarrowia lipolytica*. *Electronic Journal of Biotechnology*.

[37] Germain E., Stephenson T. (2005). Biomass characteristics, aeration and oxygen transfer in membrane bioreactors: their interrelations explained by a review of aerobic biological processes. *Reviews in Environmental Science and Biotechnology*.

[38] Chisti Y., Jauregui-Haza U. J. (2002). Oxygen transfer and mixing in mechanically agitated airlift bioreactors. *Biochemical Engineering Journal*.

